# When merchandise crowds the aisle and carts crowd the shopper: Joint effects on sales

**DOI:** 10.1371/journal.pone.0346492

**Published:** 2026-04-22

**Authors:** Mathias C. Streicher

**Affiliations:** Department of Management and Marketing, Innsbruck, Austria; MCC Boyd Tandon School of Business, INDIA

## Abstract

This research examines how fixture-induced spatial crowding suppresses in-store purchasing and how shopping aids—particularly carts—amplify this effect by expanding users’ peripersonal space and diminishing perceived control. In doing so, it addresses a central trade-off in retail design: while secondary in-aisle fixtures can increase incremental category-level exposure and sales, they simultaneously constrain shoppers’ effective space, leaving the net effect on overall aisle-level purchasing unclear. Across three studies—a 12-week scanner-based field experiment, a behavioral-tracking field experiment, and a scenario-based laboratory experiment—supported by an additional pilot, we provide causal, in-store evidence that fixture-induced spatial crowding reduces purchase behavior, lowering both in-aisle activity and sales. In one field experiment, removing mid-aisle fixtures increased sales despite reduced displayed merchandise. These effects are unlikely to be explained by differences in human crowding, assortment, or promotions. We further demonstrate that the “effective” space a shopper experiences is determined by the retail layout and the shopper’s physical footprint. Shopping aids, especially carts, expand peripersonal space and heighten sensitivity to spatial constraints, reducing perceived control. A moderated-mediation analysis shows that perceived control mediates the effects of spatial crowding on purchasing, with stronger indirect effects for cart (vs. basket) users. The results have clear implications for fixture planning by challenging the widespread assumption that maximizing the use of in-aisle fixtures supports sales at the aggregate level.

## Introduction

**Fig 1:**
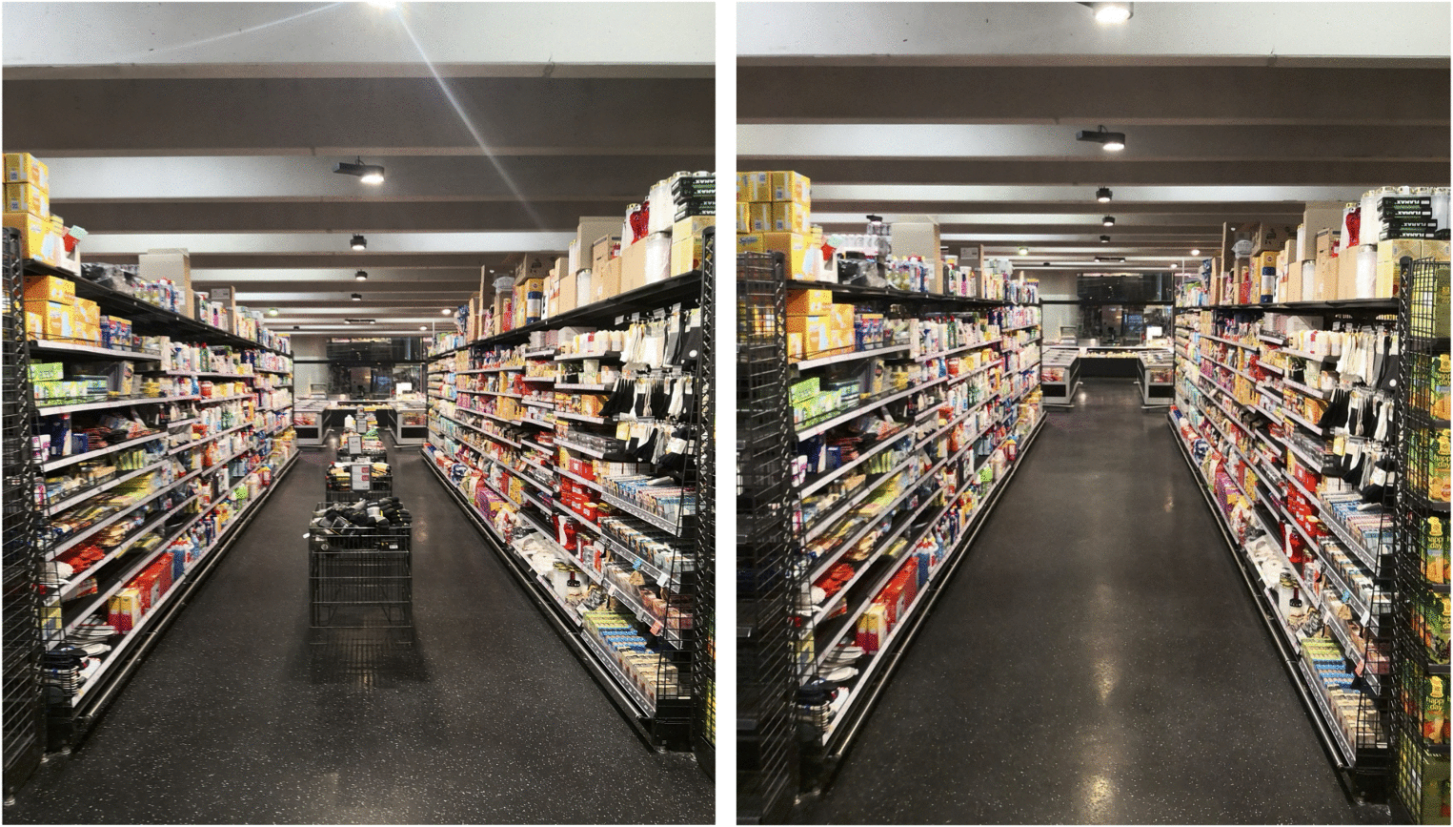
Focal aisle and manipulation (with vs. without mid-aisle floor stands).

Retail store aisles are designed to facilitate product discovery and purchasing. A widely held assumption in retailing is that increased product exposure enhances sales, particularly for unplanned purchases [1–3]. Consistent with this logic, retailers frequently place secondary displays—such as floor stands, promotional tables, or bulk bins—directly into aisles to increase merchandise visibility [4]. While such fixtures can increase exposure at the brand or category level [1,5], they also occupy available space and may constrain shoppers’ movement (see Fig 1). In-aisle fixture placement thus involves a practical trade-off that can manifest as spatial crowding [6]. Unlike human crowding, which refers to the presence and proximity of other shoppers, spatial crowding arises from the environment itself [7,8].

Two competing predictions emerge. On the one hand, secondary in-aisle fixtures can increase the amount of displayed merchandise and stimulate purchasing through additional product exposure [1]. On the other hand, the same fixtures reduce available aisle space, potentially creating a sense of spatial crowding that impedes navigation and discourages browsing and purchase behavior [6]. Whether the net effect of these competing forces is positive for overall aisle-level purchasing remains unclear.

An additional consideration is that shoppers may experience spatial constraints differently depending on how they navigate the store. Shopping aids such as carts and baskets are generally assumed to facilitate purchasing [9–11]. However, these tools also increase the shopper’s physical footprint and may alter how spatial constraints are perceived and managed during navigation [12].

Across two field experiments and one lab study, the present research investigates how fixture-induced spatial crowding influences purchase-related behavior and sales, and how this influence depends on the use of shopping aids. The results indicate that fixture-induced crowding suppresses purchase-related behavior and sales at the aisle level, with particularly strong effects for cart users.

## Conceptual background

### Spatial crowding in retail aisles and purchase behavior

Crowding is a subjective psychological state that arises when individuals perceive restrictions on their freedom of movement or action [13]. Prior research has linked spatial crowding to negative affect and avoidance tendencies [6,14,15]. In retrospective surveys, for instance, shoppers report less browsing and less adherence to purchase plans after shopping in spatially crowded stores [16]. A common source of spatial crowding is product displays inserted into aisle pathways such as floor stands. Nearly a third of all in-store displays in supermarkets are deployed in aisles [17], in part because retailers can charge brand manufacturers fees for extra shelf space (i.e., slotting fees). This extra shelf space may increase choice of the displayed products by increasing the likelihood that shoppers become aware of them [1]. In-aisle fixtures are therefore thought to contribute to overall sales as shoppers walk along an aisle and extra fixtures such as floor stands increase exposure to other products along that path [18]. Those exposures may activate new or forgotten needs [2], thereby stimulating product choice, particularly when such secondary displays are located close to their focal category’s main shelf location [5].

These studies, however, examine outcomes at the brand or category level. Hence, they do not clarify whether such in-aisle exposure strategies increase or decrease purchase behavior at the aggregate level, that is, across different products and categories located in fixture-congested aisles. Importantly, increases in brand- or category-level sales do not necessarily imply increases in overall purchasing at the aisle level. Additional in-aisle displays may shift attention and purchases toward focal brands while simultaneously increasing spatial constraints. We therefore examine aisle-level outcomes to capture the net effect of localized exposure benefits for featured items versus broader navigational costs affecting all categories in fixture-congested aisles.

Spatial crowding research suggests that reductions in aisle space caused by use of secondary displays evoke negative emotions, lower satisfaction, and ultimately reduce approach intentions [6,16,19,20]. Although prior studies have not directly observed purchase-related behavior or sales outcomes, they suggest that fixture-induced crowding may suppress purchase behavior at the aisle level. Importantly, the potential benefits of additional exposure and the costs of spatial constraint differ in scope. Exposure benefits typically arise from presentation of individual stock-keeping units and therefore manifest as brand-specific increases in sales [5]. In contrast, spatial constraints affect the aisle environment more broadly, influencing how easily shoppers can move, browse, and access products across all categories located in that aisle [6–8]. As a result, even if certain featured brands benefit from additional exposure, the navigational costs imposed by such fixture-induced crowding may suppress overall engagement with the aisle. Perceived restrictions on movement are known to reduce approach motivations and can trigger withdrawal responses that generalize beyond any single focal product [16,19,20]. Because aisle-level purchasing depends on shoppers’ willingness to browse and interact with multiple shelves, reductions in perceived navigability may have broader aggregate consequences than localized exposure effects. Gains for certain brands may therefore come at the expense of reduced purchasing elsewhere in the aisle. Taken together, although secondary displays may generate brand-specific benefits, theory suggests that the aisle-level costs of fixture-induced spatial crowding are likely to outweigh these localized exposure effects when outcomes are evaluated at the aggregate aisle level. We therefore hypothesize:

**H1:** Fixture-induced spatial crowding reduces purchase behavior

### Shopping aids, peripersonal space, and perceived control

Prior research has identified several moderators of crowding responses, including time pressure, shopping motivation, and contextual cues [6,8,21]. In contrast, shopping aids such as carts or baskets have rarely been examined in the context of spatial crowding. Specifically, shopping carts are typically assumed to facilitate purchasing by increasing carrying capacity and encouraging product acquisition [9–11]. At the same time, carts may increase spatial demand. For instance, shoppers frequently park carts to browse more freely [12], suggesting that carts may heighten perceptions of spatial crowding. A central mechanism linking crowded environments to consumer responses is perceived control—the sense that one can navigate the environment, access desired products, and make autonomous purchase decisions [22]. In retail settings, spatial features such as narrow aisles and obstructive layouts diminish perceived control and suppress approach intentions [6].

Prior research has proposed several mechanisms through which crowding affects customer responses. A recent meta-analysis integrating 73 independent samples and over 19,000 shoppers identifies perceived control, emotional responses, and store evaluation as key mediating pathways linking crowding perceptions to behavioral outcomes [23]. Importantly, emotional responses and store evaluations arise under both human and spatial crowding, whereas perceived control is more strongly implicated in spatial crowding contexts that constrain movement. Spatial crowding reliably reduces shoppers’ perceived control over their environment, whereas such effects are less consistently observed under human crowding. Perceived control therefore appears to represent a domain-specific mediator of spatial crowding, reflecting the extent to which the environment supports goal attainment by enabling shoppers to move freely and access desired products. Given that carts restrict in-aisle maneuverability [12], this raises the question of whether they also affect perceived control and approach behaviors, particularly in constrained environments.

Peripersonal space (PPS) theory provides a useful framework for understanding how shopping aids shape these effects. PPS refers to the action-oriented space near the body, represented through integrated tactile, proprioceptive, and visual cues [24,25]. Prior literature shows that tool use extends PPS, such that objects reachable with a tool are incorporated into the individual’s near-body space [26,27]. For instance, after training with a hockey stick–like tool, the typical advantage in visual detection near the hand has been shown to shift toward the tool’s functional end, indicating that the near-body action space extends beyond the biological limb [28]. Similarly, the use of prosthetic limbs in arm amputees has been found to shift the spatial boundary of tactile–auditory integration outward, suggesting that multisensory processing of environmental cues is remapped to incorporate assistive aids into the user’s effective action space [29]. Critically, research suggests that individuals react more defensively to potential violations of their near-body space, exhibiting heightened sensitivity to approaching objects within peripersonal boundaries [30].

Applied to retail environments, shopping carts can be conceptualized as temporary bodily extensions that enlarge the shopper’s PPS. In contrast, shoppers who do not use a cart—whether they carry a basket, a reusable bag, or nothing at all—maintain a smaller near-body action space. Because tool use expands PPS, carts increase the likelihood that in-aisle fixtures are experienced as intrusive. As a result, an aisle that remains objectively passable may nonetheless feel not easily navigable or comfortable when using a cart. Identical aisle layouts may thus be associated with greater losses in perceived control for cart users than for shoppers without a cart, amplifying the behavioral consequences of fixture-induced spatial crowding. Consistent with this reasoning, we hypothesize that:

**H**_**2**_ The negative effect of fixture-induced spatial crowding on purchase behavior is stronger for cart users than for shoppers not using a cart.

**H**_**3**_ Perceived control mediates the effect of fixture-induced spatial crowding on purchase behavior, and this indirect effect is stronger for shoppers using a cart than for shoppers not using a cart.

### Field study 1 – Methods

**Fig 2:**
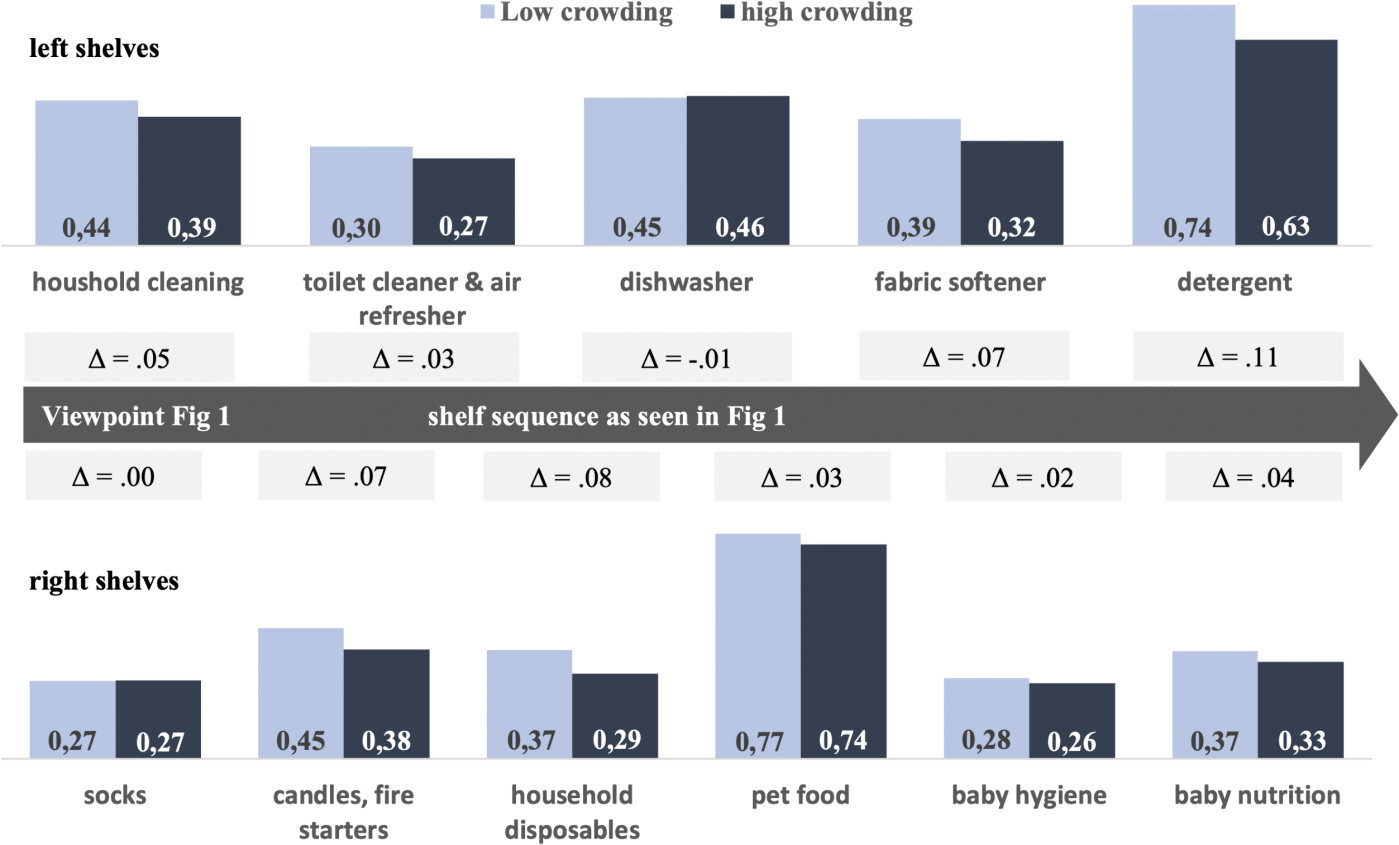
Effects of crowding on relative sales contribution in % per product category.

Study 1 provides an initial field test of whether fixture-induced spatial crowding reduces purchasing (H_1_). Using a quasi-natural experiment in a partnering supermarket (~800 m²), we compared weekly sales shares across two consecutive six-week periods: one in which a focal aisle contained five mid-aisle floor stands displaying duplicate facings from the same categories stocked in the aisle (high crowding) and one following their removal (low crowding; see Fig 1). The five floor stands were evenly distributed along the aisle. Each stand consisted of two subsections and could therefore function either as a single large display or as a mixed display for two different stock-keeping units. Across the 11 product categories located in the aisle (see Fig 2 for a comprehensive overview of categories and shelf-side placement), the floor stands displayed products from three categories on the left-hand side and four categories on the right-hand side, respectively—two fewer than the total number of categories present on each side (five on the left; six on the right).

We selected this aisle because all floor stands displayed duplicate facings located in that particular aisle—thus positioned maximally close to their home shelf locations—a context in which secondary exposure is known to be effective at generating incremental brand- or category-level sales [5]. This makes the test conservative: any negative effect of spatial crowding would emerge despite the stands being placed in a way that should, if anything, maximize their exposure benefits.

#### Design and setting.

We collected scanner-based sales data from the same focal aisle (see Fig 1). The focal aisle spanned 11 m per side and consisted of household, baby and pet staples (see Fig 2). Because these categories are staple goods, short-term seasonal fluctuations are generally less pronounced than for holiday- or weather-sensitive categories, reducing concerns that category-specific demand shocks drive the results. The intervention took place in spring 2022 and comprised two six-week periods: calendar weeks 6–11 (high crowding; five mid-aisle floor stands present) and calendar weeks 13–18 (low crowding; stands removed). Calendar week 12 served as a transition week for removing the stands and was excluded from analyses.

According to the retailer, the planogram and number of stock-keeping units for the focal aisle categories remained unchanged across the two periods. Promotions in these staple categories were predominantly long-term offers (e.g., multi-buy promotions), and no changes to planned long-term promotions were scheduled during the observation windows. Storewide opening hours and staffing followed the regular schedule. In the high-crowding condition, the clear width between a stand and the opposing shelf was approximately 90 cm; carts (~60 cm wide at the handle) could traverse the aisle under both conditions.

#### Data and measures.

Weekly POS sales share (category revenue as a percentage of total store revenue) was recorded for each of the 11 categories over 12 weeks (66 observations per condition; 132 total). Expressing category revenue relative to total store revenue reduces concerns that storewide fluctuations in traffic or demand explain condition differences, unless demand shifted disproportionately toward or away from the focal aisle categories. All data from the studies reported herein are available on the Open Science Framework: https://osf.io/yr45q/

#### Analysis.

The unit of analysis was the category revenue as a percentage of total store revenue per week. To estimate the effect of spatial crowding on category sales share, we ran a linear mixed-effects model using restricted maximum likelihood (REML) with category fixed effects and a linear time control. This specification accounts for stable differences in baseline revenue contributions across categories. The linear time control captures the week-of-month position (e.g., first, second, third, or fourth week) to account for potential intra-month fluctuations in demand across observation periods.

### Field Study 1 – Results

#### Controls.

A separate photo-based task (reported with Study 3) confirmed that the aisle with floor stands was perceived as significantly more crowded than the aisle without stands. To account for potential intra-month variation (e.g., varying demand toward or away from the focal aisle categories), we included a linear control for the week-of-month position in the main specification. The time control itself was not significant (*p* > .98), and its inclusion did not alter the direction or statistical significance of the crowding effect.

Consistent with this result, simple correlations between week-of-month position and the relative revenue contribution of the 11 focal categories were small and non-significant in both the low-crowding period (*r* = .045, *p* = .72) and the high-crowding period (*r* = −.058, *p* = .64), suggesting that week-of-month positions were not systematically associated with shifts in demand toward or away from the focal aisle categories across conditions.

#### Sales.

Consistent with H_1_, fixture-induced spatial crowding significantly reduced category sales share. Categories generated a higher share of total store revenue when mid-aisle floor stands were removed (low crowding) than when they were present (high crowding). Aggregated to the aisle, average weekly contribution rose from 4.33% under high crowding to 4.83% under low crowding—a + 0.50 percentage-point (+11.5%) uplift despite fewer displayed facings. Fig 2 reports descriptive changes in relative sales contribution for each of the 11 focal categories in shelf sequence, complementing the model-based estimates summarized in Table 1.

**Table 1:** Linear Mixed Models Predicting Relative Sales Contribution from Crowding Condition.

Predictor	*b*	*SE*	*p*
Crowding (low vs. high)	.045	.014	.002
Week (linear control)	.000	.007	.981

*Notes:* High crowding is the reference category. The dependent variable is relative sales contribution. Reported coefficients are unstandardized.

Following the aggregate trend, relative contributions increased for the majority of categories when mid-aisle fixtures were removed (9 of 11), with one category showing no change and one a marginal decrease. Of the 11 focal categories, four (household disposables, baby nutrition, toilet cleaner & air refresher, and fabric softener) were not featured on the mid-aisle floor stands in the high-crowding condition. Relative sales contributions for all four of these non-featured categories increased following removal of the stands. At the same time, categories that were featured on the floor stands exhibited a mixed pattern, with most increasing but one showing no change and one a marginal decrease. Both featured and non-featured categories exhibited positive mean changes in relative sales contribution following removal of the mid-aisle stands (mean Δ_featured_ = +3.9 percentage points; mean Δ_non-featured_ = +5.5 percentage points), suggesting that the aggregate uplift was not limited to categories directly affected by duplicate exposure.

**Fig 3:**
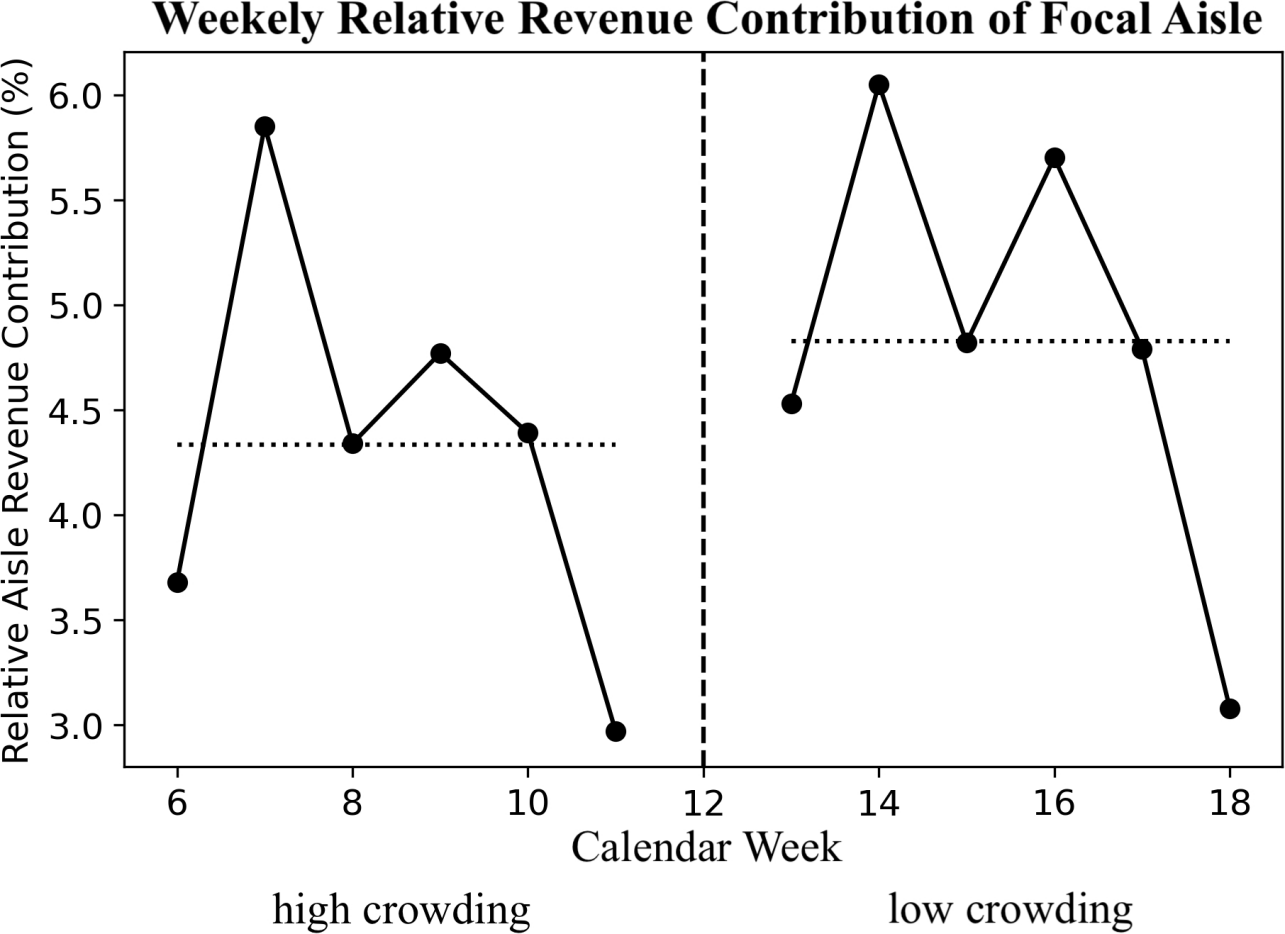
Weekly relative revenue contribution of the focal aisle categories across the 12-week observation window. The dashed vertical line indicates the transition week. Dotted horizontal lines indicate period means.

This absence of a consistent pattern based on whether a category was featured on a floor stand is consistent with an aisle-level effect rather than category-specific exposure benefits or losses. Fig 3 displays the weekly relative revenue contribution of the focal aisle categories across the full observation window, with a visible upward shift following removal of the mid-aisle fixtures.

Because the analysis pools multiple product categories with heterogeneous baseline sales levels, we conducted additional robustness checks to ensure that the estimated effect was not driven by any single category. Specifically, we re-estimated the model repeatedly while excluding one category at a time. Across these leave-one-category-out specifications, the estimated crowding effect ranged from *b* = .037 to *b* = .052 and remained statistically significant in all cases, indicating that the result is not attributable to idiosyncratic movements in any particular category.

Together, these analyses support the robustness of the main finding that fixture-induced spatial crowding suppresses sales at the aisle level (H_1_). Results are consistent with model-free descriptive patterns shown in Fig 2 and Fig 3—including positive mean uplifts for both categories displayed on the mid-aisle floor stands and those not displayed— and are robust to alternative specifications, including models with nonlinear time trends and leave-one-category-out analyses. At the same time, sales data precludes disentangling shopper types. Study 2 therefore moves to in-store tracking to test individual-level purchase behavior and moderation by shopping aid (cart vs. basket).

### Field study 2 – Methods

Study 2 builds on the aggregate sales effect observed in Study 1 by testing H_2_. Specifically, it examines whether fixture-induced spatial crowding reduces stop-level purchase consideration in a naturalistic setting and whether this effect is moderated by shopping aid (cart vs. no cart). We recorded how often shoppers physically handled merchandise while stopping in the aisle. Product handling reflects purchase consideration and approach behavior and provides a behavioral proxy for purchasing, as touching or examining a product typically precedes purchase and is strongly associated with subsequent product choice [31,32].

#### Design and setting.

The study took place in the same target aisle as Study 1, using the identical manipulation of mid-aisle floor stands (present = high crowding; removed = low crowding) while holding aisle-related store activities constant. Data were collected between 10:00 a.m. and 5:00 p.m. on six weekdays (Monday, Wednesday, Friday across two consecutive weeks). In week 1 (*n* = 280), floor stands were present; in week 2 (*n* = 298), they were absent. Fig 1 illustrates the two conditions.

#### Participants and procedure.

During the observation window, 578 shoppers entered the aisle. Observations were covert, and no personal or demographic data were collected. Two trained research assistants were stationed unobtrusively at opposite ends of the aisle. Each observer tracked and coded the behavior of a single shopper at a time from aisle entry to exit. When multiple shoppers entered the aisle simultaneously, observers coded different shoppers in parallel to avoid missed observations; the same shopper was never coded by more than one observer.

#### Measures.

Unbeknownst to the shoppers, we observed two key behaviors: (i) the number of times a shopper stopped at a shelf serving as an indicator of emerging interest, and (ii) the number of instances in which they touched or examined products as an indicator of purchase consideration. Additionally, the experimenters recorded whether shoppers used a shopping cart or not (with “not” including use of a basket, carry bag, or no carrying aid), and whether they were accompanied by a companion, as the presence of companions could theoretically contribute to perceived crowding.

#### Analysis.

Following standard practice, extreme outliers on unbounded count variables were identified via the Tukey criterion and winsorized to the nearest non-outlier [33,34]. Results were robust with or without winsorization. Because both outcomes are overdispersed (i.e., variance > mean), we estimated negative binomial regressions (log link, estimated dispersion). Predictors included spatial crowding (1 = high, 0 = low), cart use (1 = cart, 0 = no cart), and their interaction. Parallel analyses with number of stops as the dependent variable are reported in S1 Appendix.

## Study 2 – Results

### Controls

Store-level data indicated comparable shopper traffic across conditions, with no difference in total checkouts (2,099 with stands vs. 2,061 without; χ², *p* = .62) or in the number of aisle entrants (*p* = .56). Among observed shoppers, the probabilities of being accompanied (*p* = .17) and of using a cart (38% overall; *p* = .56) were also comparable across conditions. Together, these indicators suggest that shopper traffic and composition did not systematically differ between conditions, reducing concerns that human crowding or shopper characteristics account for the observed effects.

### Purchase consideration

**Fig 4:**
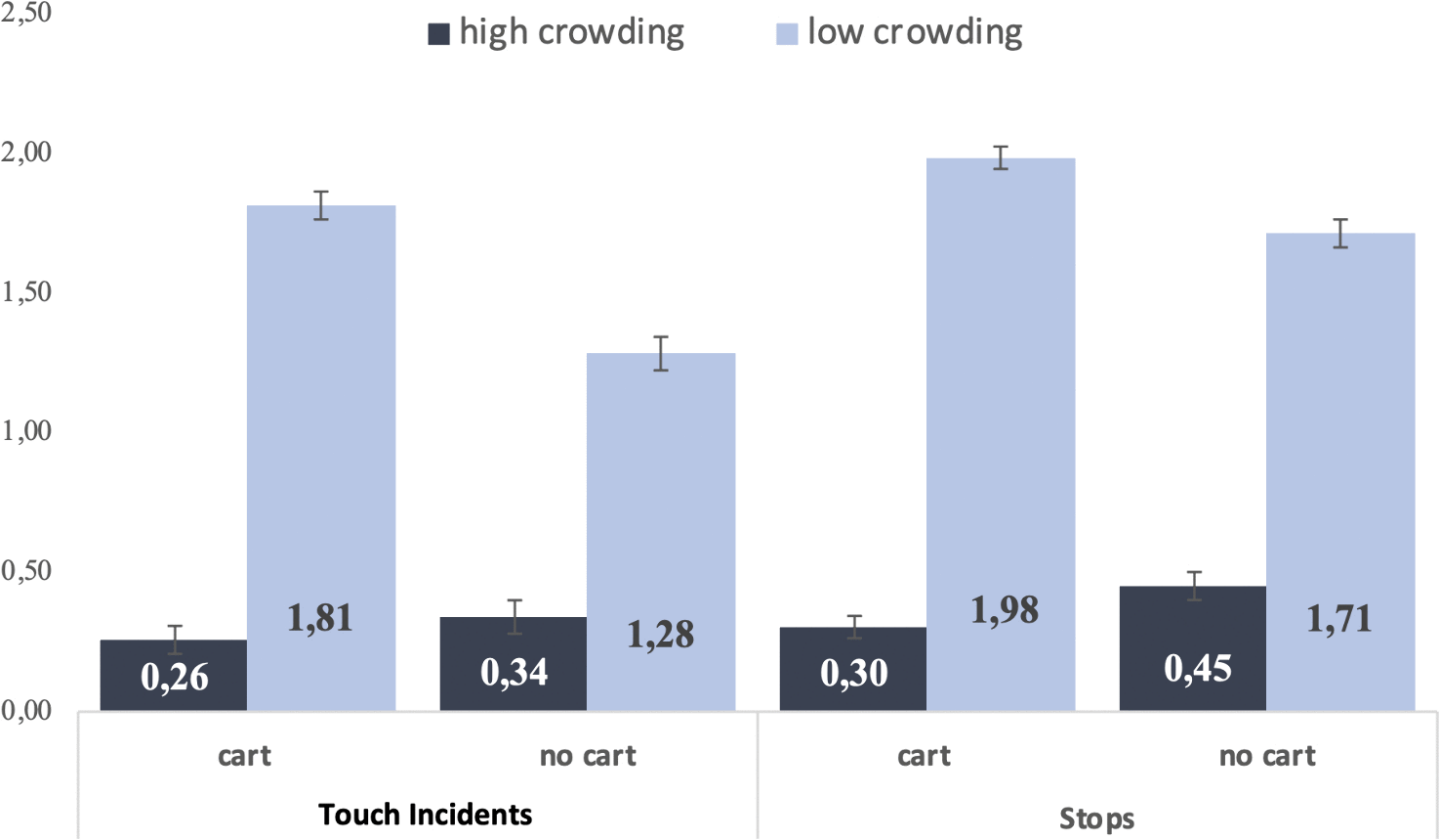
Mean Touch and Stops Study 2. Error bars indicate ± 1 *SE.*

The regression predicting product-touch events was significant overall, χ²(3) = 229.31, *p* < .001. There was a main effect of crowding (Wald χ²(1) = 173.20, *p* < .001): shoppers in low crowding handled and examined significantly more products than shoppers in high crowding. The incidence-rate ratio indicated that, holding cart use constant, shoppers in low (vs. high) crowding engaged in approximately 7.05 times as many product-touch events. The main effect of cart was not significant (Wald χ²(1) = 1.46, *p* = .23). Importantly, the crowding × cart interaction was significant (Wald χ²(1) = 6.22, *p* = .01; B_exp_ = 0.54). This indicates that low crowding benefitted cart users more strongly: cart users engaged in roughly 7.05 × more touch incidents in low vs. high crowding, whereas non-cart users exhibited about 3.81 × more (7.05 × 0.54 ≈ 3.81). Importantly, the results are unlikely to be attributable to differences in human crowding or shopper composition, as store traffic, cart usage, and accompaniment rates were comparable across conditions. Fig 4 shows outcomes for both stops and touch events.

Parallel analyses with number of stops as the dependent variable yielded the same pattern (main effect of crowding, significant interaction with cart). Full results appear in S1 Appendix. Together, the results support H_2_ by showing that shopping aids moderate behavioral responses to spatial crowding. Study 3 next examines the psychological mechanism underlying these effects by isolating perceived control as a mediator of spatial crowding responses.

### Study 3 – Methods

Study 1 showed that fixture-induced spatial crowding depresses sales in-store, and Study 2 demonstrated stronger behavioral effects for cart users, consistent with moderation by shopping aid. Study 3 examines the underlying mechanism: whether spatial crowding reduces perceived control, and whether this reduction—and its downstream impact on purchase behavior—is stronger for carts than baskets (H_3_). The purpose of this study is to isolate the underlying psychological mechanism rather than to estimate the magnitude of behavioral or sales effects observed in the field. Before testing the full moderated-mediation model, we conducted a brief pilot study to validate the photo-based procedure of Study 3.

### Pilot study

To assess the peripersonal space account underlying H_3_ and to prepare the main mechanism test in Study 3, we conducted a pilot study using an “aisle ruler” task. The pilot served two purposes. First, it assessed whether static, photo-based stimuli can reliably elicit perceptions of navigational constraint, despite the absence of actual movement. Second, it provided preliminary evidence that shopping carts systematically alter spatial perception—an effect that is subsumed by H_3_ but has not been directly demonstrated in this context.

Two hundred twenty-eight participants were randomly assigned to physically hold either a shopping cart or a basket in a laboratory setting while viewing a picture of the focal aisle used in Studies 1 and 2 and later employed in Study 3, with mid-aisle floor stands present (i.e., high crowding). Participants first rated their perceived navigational freedom in response to the congested aisle. They then completed a computer-based task in which the aisle could be incrementally widened until they reached their preferred width for comfortable navigation with the assigned shopping aid.

Consistent with peripersonal space expansion, cart users reported significantly lower perceived navigational freedom and selected significantly wider aisle widths than basket users (Δ = 3.77%). These results indicate that shopping carts increase sensitivity to spatial constraints in aisles, motivating the crossed manipulation of spatial crowding and shopping aid in Study 3 using a photo-based task. Full procedural details and additional analyses are reported in S2 Appendix.

### Participants

**Fig 5:**
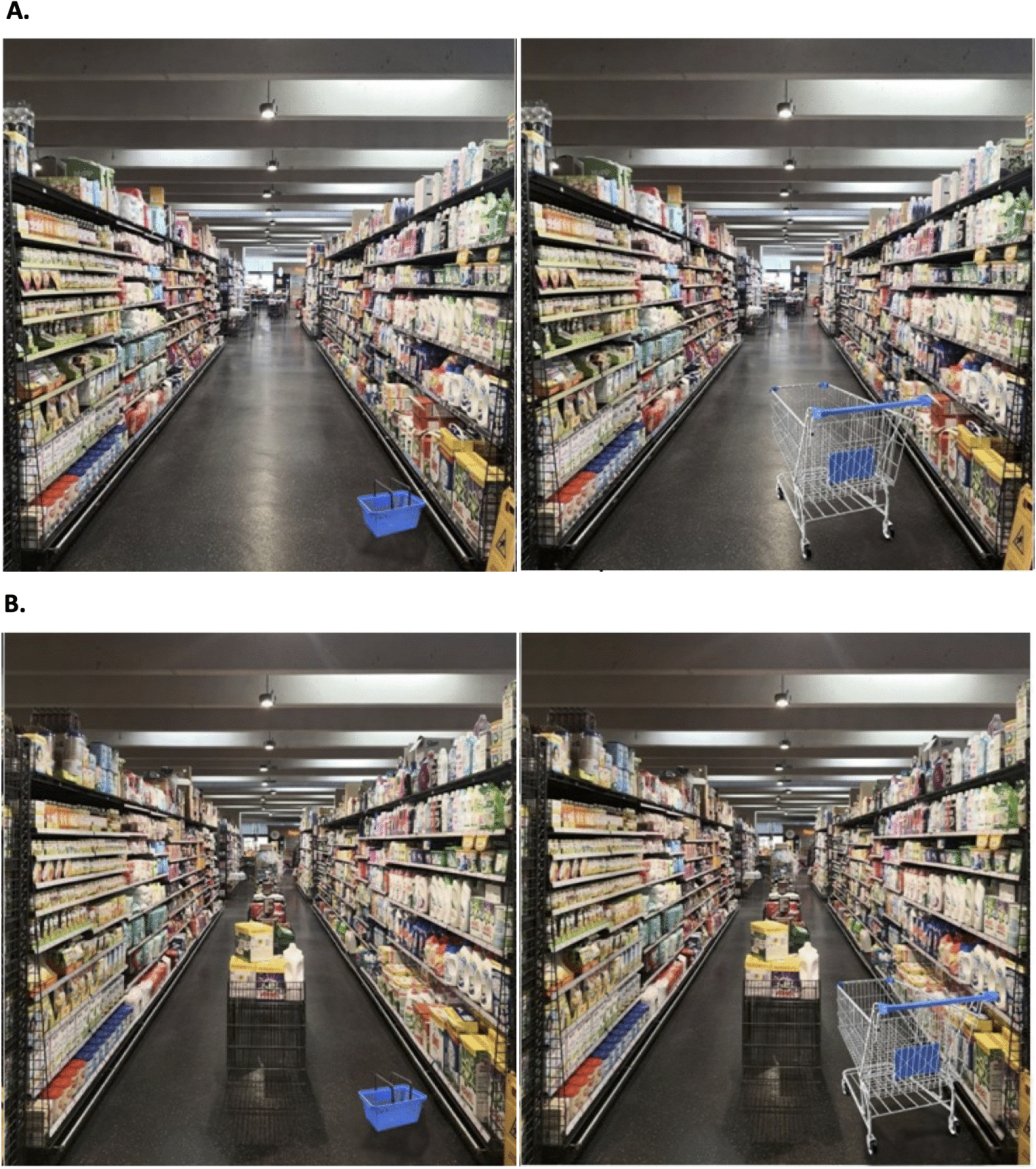
Crowding scenarios Study 3.

We recruited 200 participants via Prolific (mean age = 43 years; 66.5% female, 33% male, 0.5% diverse), who participated for compensation consistent with Prolific guidelines. Informed consent and an attention check preceded the study. The experiment was conducted online using photo stimuli of the same store aisle as in Studies 1–2. Participants were randomly assigned to low- vs. high-crowding conditions (between subjects). Within each condition, they completed two trials in randomized order: imagining navigating the aisle with a basket and with a cart. After each trial, participants reported perceived control and shopping intentions. Stimuli were scaled so both aids realistically fit the aisle under both crowding conditions (Fig 5). The session concluded with a manipulation check (“How spatially crowded did the aisle look to you?” 1–7). Full item wordings appear in Table 2.

**Table 2:** Measures and Items used in Study 3.

Perceived Control (modified from [6]; basket *α* = .93; cart *α* = .95)
I rarely feel stuck or restricted while shopping here; I feel in control of the situation in this aisle; Shoppers can easily navigate to the items they need; The customer is in control in this aisle. 1 = strongly disagree; 7 = strongly agree
**Shopping Intention** (modified from [35])
How likely are you to shop in this aisle? 1 = not at all likely; 7 = very likely
**Perceived Crowding** (ad hoc)
How spatially crowded did the aisle look to you? 1 = not at all crowded; 7 = very crowded

## Study 3 – Results

### Controls

The manipulation was successful: participants rated the aisle with floor stands as significantly more crowded (*M* = 6.06) than without (*M* = 3.36), *t*(198) = 11.66, *p* < .001. Age and gender did not differ across conditions (*p*s > .16 and >.59).

### Perceived control

**Fig 6:**
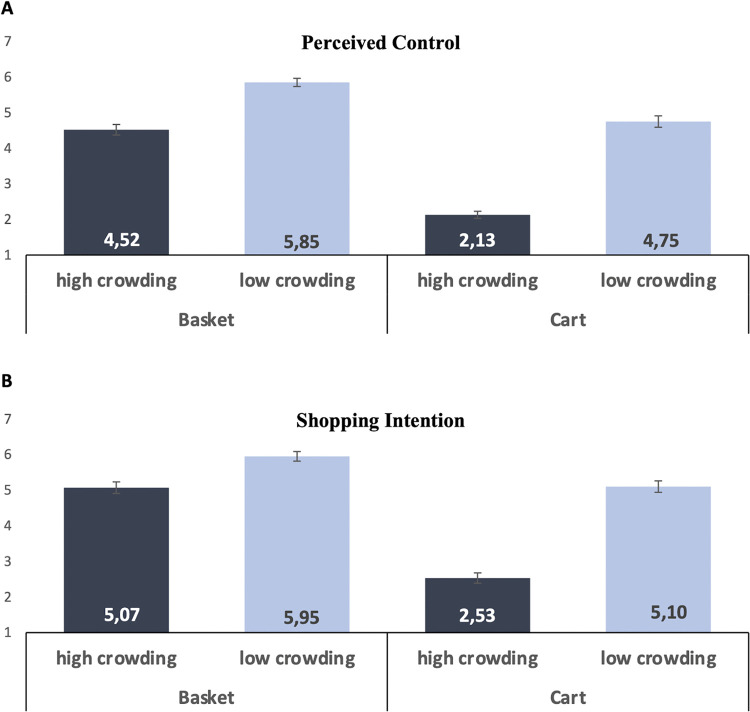
Effects of crowding and shopping aid on perceived control (A) and shopping intention (B). Error bars indicate ± 1 *SE.*

A mixed-model ANOVA with crowding (between: low vs. high) and shopping aid (within: cart vs. basket) revealed main effects of aid, *F*(1, 198) = 243.05, *p* < .001 (lower control with cart than basket; see Fig 6A), and crowding, *F*(1, 198) = 169.10, *p* < .001 (lower control under high crowding; see Fig 6A), as well as a significant interaction, *F*(1, 198) = 33.20, *p* < .001. Follow-up tests showed that perceived control was lower under high vs. low crowding for both carts (*M* = 2.13 vs. 4.75; *t*(198) = 13.94, *p* < .001, *d* = 1.93) and baskets (*M* = 4.52 vs. 5.85; *t*(198) = 7.07, *p* < .001, *d* = 1.01), wi*t*h a larger decrement for carts (Fig 6A).

### Shopping intentions

A parallel ANOVA revealed main effects of aid, *F*(1, 198) = 165.10, *p* < .001 (lower intentions with cart; see Fig 6B), and crowding, *F*(1, 198) = 104.77, *p* < .001 (lower intentions under high crowding; see Fig 6B), plus a significant interaction, *F*(1, 198) = 40.87, *p* < .001. Follow-ups confirmed lower intentions under high vs. low crowding for both carts (*M* = 2.53 vs. 5.10; *t*(198) = 11.74, *p* < .001, *d* = 1.57) and baskets (*M* = 5.07 vs. 5.95; *t*(198) = 4.11, *p* < .001, *d* = 0.59), wi*t*h the larger effect for car*t*s (Fig 6B).

### Moderated mediation

To estimate the paths in our moderated-mediation model with appropriate subject-level dependence, we first fit a linear mixed model (LMM) with random intercepts for participant. On the *a*-path (crowding → perceived control), effects of crowding (*B* = −1.48, SE = 0.11, *t* = 13.01, *p* < .001), shopping aid (*B* = −1.32, SE = 0.08, *t* = 15.59, *p* < .001), and *t*heir interaction (*B* = −0.71, SE = 0.12, *t* = 5.77, *p* < .001) were significant. On *t*he *b*-path (perceived control → shopping intentions), perceived control was a strong positive predictor (*B* = 0.55, *SE* = 0.05, *t* = 11.91, *p* < .001), while a residual direct effect of crowding remained (*B* = −0.47, SE = 0.09, *t* = 5.22, *p* < .001), indicating complementary mediation [36].

Because standard LMM implementations do not compute bootstrapped conditional indirect effects, we replicated the model using PROCESS, Model 7 [37] with 10,000 bootstrap samples to obtain bias-corrected confidence intervals for the conditional indirects. The indirect effect of crowding via perceived control was significant for both carts (*B* = −0.95, 95% CI [−1.28, −0.64]) and baskets (*B* = −0.61, 95% CI [−0.81, −0.42]); the total indirect effect was significant (*B* = −0.78, 95% CI [−1.00, −0.57]). Crucially, the contrast of conditional indirect effects was significant (*B* = −0.34, 95% CI [−0.57, −0.10]), indicating a stronger mediated effect for carts than for baskets. Study 3 demonstrates that perceived control mediates the effect of spatial crowding on shopping intentions and that this indirect effect is amplified for cart users. While mediation is significant for both aids, the larger indirect effect for carts supports the moderated-mediation account in H_3_.

## General discussion

This research was motivated by a fundamental tension in retail layout design. On the one hand, retailers frequently prioritize product exposure, often explicitly trading off aisle space for additional displays. Prior research shows that such secondary placements—especially when located close to a product’s home shelf—can activate needs and generate incremental sales at the brand or category level. On the other hand, spatial crowding research suggests that constraining aisle space by in-aisle fixtures may frustrate shoppers and suppress approach behavior. Which of these countervailing tendencies dominates at the aggregate aisle level has remained an open empirical question.

Across three complementary studies, we provide convergent evidence that, in this trade-off, the costs of spatial crowding can outweigh the benefits of additional exposure. Importantly, we evaluate these outcomes at the aggregate aisle level, where localized exposure benefits for some products may coincide with broader navigational costs affecting all shoppers navigating the aisle. Study 1 shows that removing mid-aisle fixtures increased relative aisle sales despite fewer displayed facings. Descriptive category-level analyses further indicated positive mean uplifts for both categories displayed on the floor stands and those not displayed, suggesting that the observed effect reflects a broader aisle-level response rather than localized exposure gains or losses. Study 2 replicates this pattern behaviorally in the same store: crowded layouts reduced in-aisle stopping and product-handling behaviors that typically precede purchase, with significantly stronger effects for shoppers using carts. The pilot study for Study 3 further indicates that shoppers physically holding carts (vs. baskets) prefer wider aisles and report lower perceived navigational freedom in response to crowded aisle conditions. Study 3 then isolates the underlying mechanism, demonstrating that fixture-induced crowding more strongly reduces perceived control and shopping intentions for imagined cart (vs. basket) use, consistent with a moderated-mediation account.

Importantly, these effects emerge in a conservative test context. In Studies 1–2, the focal aisle’s assortment, planograms, and long-term promotional programs were held constant across conditions, and Study 2 indicates that changes in human crowding or shopper composition are unlikely to explain the results (comparable store traffic, aisle entry, accompaniment, and cart usage across conditions). Moreover, the stands displayed products from the focal aisle and were positioned close to home shelf locations, a placement known to enhance exposure effects [5]. That negative effects nonetheless emerged indicates that fixture-induced spatial crowding can suppress purchasing even under conditions designed to maximize exposure benefits.

Taken together, the findings are consistent with a mechanism in which spatial constraints undermine perceived control, reduce engagement, and ultimately depress aisle-level purchase behavior. By integrating scanner data, field tracking, and controlled experiments, our findings extend the crowding literature by showing that spatial crowding affects not only subjective experiences but also commercial outcomes [6,16,19,20]. The results also suggest that shopping aids contribute to spatial crowding: carts appear to increase sensitivity to spatial constraints, consistent with a peripersonal space account in which tools expand near-body action space and heighten responses to intrusions [23–26]. In practical terms, layouts that remain objectively passable may still be experienced as difficult to navigate when using a cart, amplifying perceived control losses and downstream behavioral effects.

### Practical relevance

The results imply that evaluating in-aisle fixtures solely on brand- or category-level performance may be incomplete when fixtures meaningfully constrain navigational space. Retailers may therefore benefit from assessing exposure-related gains alongside aisle-level outcomes and shopper experience. Where reducing in-aisle congestion is feasible, reducing fixture-related obstructions may improve navigability—especially in stores with high cart usage—while preserving exposure through alternative placements such as endcaps or less intrusive secondary placements [5].

## Limitations and future directions

Our focus on spatial (not human) crowding was deliberate for clarity. Future research could examine whether human crowding produces similar amplification effects for cart users and whether combined spatial and social density imposes additional costs. Second, our manipulation contrasted the presence versus absence of mid-aisle displays. Prior research has demonstrated that secondary placements can generate exposure-related gains for featured brands or categories [1,5], and our findings should not be interpreted as implying that such displays are ineffective per se. Rather, our results highlight that when deployed within constrained aisle environments, the navigational costs associated with fixture-induced spatial crowding may outweigh localized exposure benefits at the aggregate aisle level. Managers often face limited degrees of freedom in reducing secondary displays in aisles. This leaves open for future investigation whether intermediate levels or configurations of in-aisle fixtures can simultaneously preserve exposure benefits while mitigating congestion-related losses in perceived control and purchase outcomes.

Third, the quasi-experimental setting of Study 1 entails limitations with respect to promotional activity. The retailer confirmed that planned long-term promotional programs (e.g., multi-buy offers) for the focal aisle categories remained unchanged across the observation windows. However, we cannot exclude the possibility that short-term promotional activities (e.g., temporary price discounts) affecting individual products within the aisle occurred during the study period. While our primary outcome measure expresses category revenue relative to total store revenue—thereby scaling aisle-level demand by overall purchasing activity—such time-varying promotions would need to systematically coincide with the intervention period and disproportionately affect multiple focal categories to account for the observed aggregate shift. Nonetheless, future studies could more directly track promotional activity or employ matched control stores to more cleanly isolate such effects.

Fourth, the before–after design of Study 1 does not allow us to fully rule out broader seasonal or period-specific influences. Although the focal categories are staple goods and relative sales share was used to scale aisle-level demand by overall store purchasing activity, future research could employ control stores or historical pre-period data to more precisely estimate causal effects. Fifth, in-store secondary placements often generate manufacturer payments (e.g., slotting fees) that are not reflected in aisle-level sales outcomes. As our study focuses on purchase-related behavior and revenue contribution rather than store profitability, future research could investigate the fee levels required to offset potential congestion-related reductions in aisle-level purchasing. Lastly, contexts with large shopping aids (e.g., warehouse clubs) pose distinct spatial challenges, and certain store types activate more hedonic while others activate more utilitarian shopping orientations [38]—all factors that may moderate the effects of fixture-induced spatial crowding.

## Supporting information

S1 Appendix Results of Stops, Study 2. (DOCX)

S2 Appendix Pilot study. (DOCX)
