# Supplementary material for: When merchandise crowds the aisle and carts crowd the shopper: Joint effects on sales
Source: PLoS One. 2026 Apr 22;21(4):e0346492. doi: 10.1371/journal.pone.0346492 (PMC13102192; doi:10.1371/journal.pone.0346492)
Supplement: S1 Appendix — (DOCX) [file pone.0346492.s001.docx]

**S1 Appendix: Results of Stops, Study 2**

Similar to the count data of products being touched, shoppers’ stops were characterized by a high frequency of zeros, resulting in strongly skewed and overdispersed count distributions (i.e., variance > mean). To account for these distributional properties, we used a negative binomial regression with a log link function and an estimated dispersion parameter to examine the effects of crowding (1 = crowded, 0 = uncrowded), shopping cart use (1 = cart, 0 = no cart), and their interaction on the number of times shoppers stopped to examine products.

The model was significant overall, *χ*²(3) = 253.99, *p* < .001, indicating that the predictors explained a significant portion of variance in the outcome. There was a significant main effect of crowding, Wald χ²(1) = 201.40, *p* < .001. Shoppers in the uncrowded condition (i.e., without floor stands) physically explored significantly more items than those in the crowded condition. The exponentiated coefficient, *B*ₑₓₚ = 6.55, indicates that, holding cart use constant, assortment exploration in the uncrowded condition was over 6.55 times higher than in the crowded condition. No significant main effect of cart use emerged, Wald χ²(1) = 1.14, *p* = .29. However, a significant interaction between crowding and cart use was observed, Wald χ²(1) = 6.00, *p* = .01. The interaction (Bₑₓₚ = 0.58) reveals that the positive effect of an uncrowded environment on assortment exploration was stronger for shoppers with carts than for those without carts.

To illustrate, shoppers with carts explored approximately 6.55 times more items in the uncrowded compared to the crowded condition. In contrast, shoppers without carts explored only about 3.80 times more items in the uncrowded than in the crowded condition (6.55 × 0.58 ≈ 3.80). Thus, spatial crowding led to significantly fewer stops among cart users than non-cart users.
