# Supplementary material for: When merchandise crowds the aisle and carts crowd the shopper: Joint effects on sales
Source: PLoS One. 2026 Apr 22;21(4):e0346492. doi: 10.1371/journal.pone.0346492 (PMC13102192; doi:10.1371/journal.pone.0346492)
Supplement: S2 Appendix — (DOCX) [file pone.0346492.s002.docx]

**S2 Appendix: Pilot study**

The goal of the pilot study is to provide a behavioral test of peripersonal space expansion. Using an “aisle ruler” paradigm, we assess whether shoppers with a cart as compared to those with a basket prefer wider aisle widths in a spatially crowded setting. If cart use expands the user’s functional interaction space, then cart users should report greater spatial demand even when no real movement is required. The shopping aid served as the independent variable, with two outcome measures: preferred aisle width and perceived control.

**Methods**

**Participants.** A total of 228 participants (mean age = 23 years; 55% female) were recruited in the lobby of a European university. Participation was voluntary, with informed consent obtained prior to the study. Participants were randomly assigned to one of two conditions (i.e., shopping cart versus shopping basket).

**Procedure.** Participants were tested individually in a controlled setting. Upon arrival, each participant was handed either a standard shopping cart or a hand-held basket, identical to those used in the partnering retailer’s stores. After briefly holding their assigned shopping aid, they were seated in front of a 21-inch monitor and shown an image of a crowded store aisle. To simulate realistic spatial constraints, the image depicted a high crowding configuration with mid-aisle floor stands commonly found in retail environments (see S1 Fig, Step 1). First, participants completed a two-item measure of perceived navigational freedom, assessing the extent to which the initial aisle width allowed for unobstructed movement (i.e., “Customers can move freely in this aisle”; “I can easily switch sides in this aisle”; r = .81, p < .001), rated on a 7-point scale (1 = strongly disagree, 7 = strongly agree). This measure specifically targeted *navigational control* which is the extent to which the physical space allows shoppers to move freely within the aisle (van Rompay et al., 2008).

Participants then completed a self-paced, computer-based “aisle ruler” task designed to assess their preferred aisle width for comfortable navigation with their assigned shopping aid. The visual stimulus was constructed from the original aisle photo, which was decomposed into three components: the left shelf section, right shelf section, and central floor stand area. Using these elements, we created 20 incremental images, each representing a slightly wider version of the aisle. In each successive step, the left and right shelves were shifted outward by one unit, resulting in a 2.4% increase in total aisle width per step (see S1 Fig). To preserve visual realism, the blank space created by this expansion was filled with duplicate floor textures from the original image, producing a seamless appearance.

These 20 images were arranged in a PowerPoint presentation, with one slide per image. Pressing the Enter key advanced the presentation by one step, creating the visual illusion of the aisle gradually widening. Participants were instructed to continue until they reached the aisle width, they considered ideal for navigating the aisle with their assigned shopping aid.

Participants could adjust the aisle width up to 20 steps or return to a previous step using the arrow keys. If they reached the final slide, they were asked to return to the version that best reflected their preferred aisle width. The final step selected served as a behavioral indicator of the participant’s space requirement. Finally, they also reported their age and gender.

**S2 Fig. 1 Aisle Ruler Visualization in Pilot study: Step 1 vs. Step 10 (24% Expansion)**

**
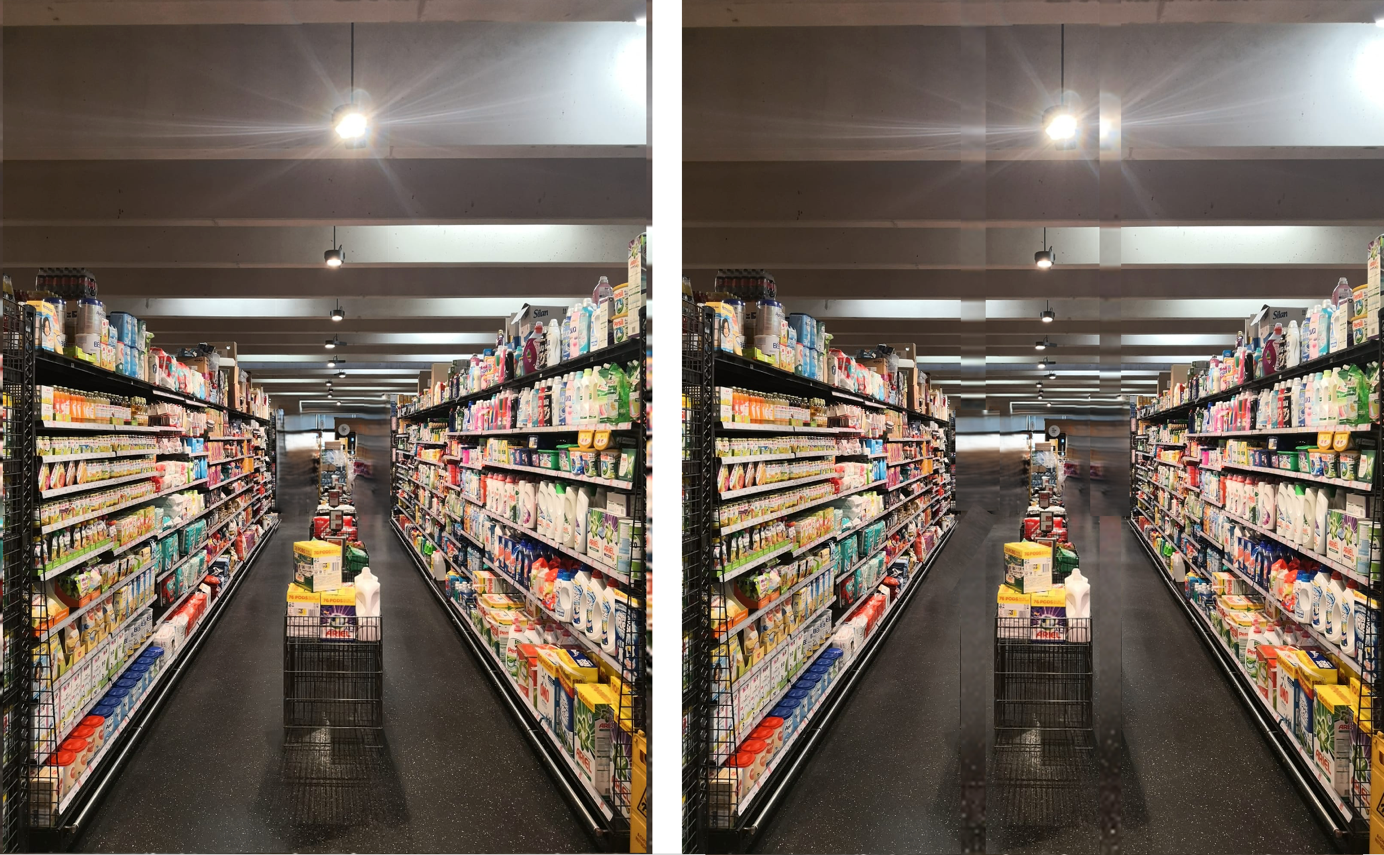
**

**Results**

**Controls.** Independent-samples t-test confirmed no significant difference in age across conditions (*p* = .34). A chi-square test revealed no significant difference in gender distribution (*p* = .11). The two groups were thus matched for demographic variables.

**Perceived navigational control**. An independent-samples t-test revealed a significant difference in perceived navigational freedom as a function of shopping aid, *t*(226) = 2.08, p = .039; *d* = 0.28. Participants assigned to the cart condition reported significantly lower perceived navigational freedom (*M* = 4.11, *SD* = 1.69) compared to those in the basket condition (*M* = 4.56, *SD* = 1.55). Results are illustrated in S2 Fig.

**Preferred aisle width**. An independent-samples t-test revealed a significant difference in preferred aisle width as a function of shopping aid, *t*(226) = 2.53, *p* < .05, *d* = 0.34. Participants who held a shopping cart selected wider aisles (*M* = 14.20 steps, *SD* = 4.87) than those holding a basket (*M* = 12.63 steps, *SD* = 4.50). As each step corresponded to a 2.40% increase in aisle width, cart shoppers prefer the aisle to be 3.77% wider than basket shoppers’ desire.

**S2 Fig. 2 Effects of shopping aid on spatial demand and control. Error bars indicate ± 1 *SE***

**Mediation.** To further examine whether the observed reduction in perceived navigational freedom helps explain the increased spatial demand, we conducted an exploratory mediation analysis (Hayes, 2017; Model 4, 10k bootstraps). Specifically, we examined whether perceived navigational freedom of the aisle in the initial step 1 (i.e., narrowest aisle configuration) mediates the relationship between shopping aid (0 = basket, 1 = cart) and the aisle width preference (i.e., as a proxy for peripersonal expansion).

As indicated above, shopping aid significantly predicted perceived navigational freedom (path a), *B* = 1.57, *SE* = 0.62, *t*(226) = 2.53, *p* = .01, such that cart users reported lower perceived freedom than basket users. Perceived control, in turn, significantly predicted aisle width preference (path b), *B* = –.09, *SE* = .02, *t*(225) = 4.18, *p* < .001, such that lower perceived freedom was associated with greater space demand.

The direct effect of shopping aid on space demand was nonsignificant when accounting for aisle preference (path c′), *p* = .15. Most importantly, the indirect effect was significant, *B* = –.15, *SE* = .07, 95% *CI* [–0.31, –0.03], revealing indirect-only mediation (Zhao et al., 2010).

**Discussion**

The pilot study provides a behavioral test of the theoretical assumption that shopping aids such as carts lower perceived navigational freedom and expand consumers’ space demand, even in the absence of actual movement. Critically, shopping carts relative to baskets reduced perceived control, a key behavioral response to spatial crowding (Hui & Bateson, 1991). Mediation analysis confirmed that cart-induced increases in space demand were not direct but occurred via lower perceived navigational freedom. This supports the proposed mechanism that increasing the physical footprint increases boundaries of peripersonal space (i.e., space demand) via greater sensitivity to spatial violations (i.e., perceived control). These findings establish a proximal mechanism that helps explain why shoppers using carts are particularly sensitive to the impact of fixture-induced crowding. By isolating this process in the absence of layout variation, the pilot study provides additional confidence for the predicted moderated mediation of hypothesis 3.
